# Epidemiology of human papillomavirus related cancers in India: findings from the National Cancer Registry Programme

**DOI:** 10.3332/ecancer.2022.1444

**Published:** 2022-09-07

**Authors:** Thilagavathi Ramamoorthy, Krishnan Sathishkumar, Priyanka Das, Kondalli Lakshminarayana Sudarshan, Prashant Mathur

**Affiliations:** Indian Council of Medical Research – National Centre for Disease Informatics and Research, Nirmal Bhawan-ICMR Complex (II Floor), Poojanahalli, Kannamangala Post, Bengaluru-562 110, Karnataka, India

**Keywords:** human papillomavirus, cervical cancer, oropharyngeal cancer, India, epidemiology

## Abstract

Human papillomavirus (HPV) causes more than one-fourth of infection related cancers globally. The present study summarises the epidemiology of HPV related cancers in India, with a special focus on cervical and oropharyngeal cancer, utilising the National Cancer Registry Programme (NCRP) data. The data on HPV related cancer incidence and treatment were extracted from 28 population-based and 96 hospital-based cancer registries under the NCRP network. Incidence was presented as rates, clinical extent of disease and treatment provided as percentages. Joinpoint regression analysis was performed to calculate annual percent change in age adjusted incidence rates (AARs) over time. Incidence of HPV related cancers for 2025 was projected. Among all cancers, 7.5% were HPV related cancers. Cervical cancer (87.6%) and oropharyngeal cancer (63.2%) were the most common HPV related cancers in India among females and males, respectively. Cervical cancer was highest in Papumpare district (AAR: 27.7 per 100,000) and oropharyngeal cancer among males in East Khasi Hills district Population Based Cancer Registry (AAR: 11.4 per 100,000). In most PBCRS, cervical cancer incidence rate decreased significantly over the period of time. The majority of these cancers presented at locoregional spread stage of the disease and were treated with chemoradiation. The projected incidence of HPV related cancers is expected to increase to 121,302 by 2025. Implementation of effective prevention and control strategies like HPV vaccination and scaling up of screening could reduce the burden of HPV related cancers. Evidence from NCRP serves as the baseline to monitor the impact of HPV related policies and programmes in improving the outcome and prognosis.

## Introduction

Globally in 2018, human papillomavirus (HPV) accounted for 31% cancer cases amongst 13% of all human infection related incident cancer cases [1]. HPV types 16, 18, 26, 31, 33, 35, 39, 45, 51, 52, 53, 56, 58, 59, 66, 68, 73 and 82 are classified as carcinogenic to humans [2]. Persistent HPV infection (especially HPV types 16 and 18) is known to cause the cervical cancers and a significant proportion of the anogenital and head and neck cancers [3]. Among HPV attributable cancers, 80% are cervical cancer which are preventable through HPV vaccination along with other HPV related cancers [4]. In 2018, World Health Organization has called for a global action towards elimination of cervical cancer (a threshold of 4 per 100,000 women-year) and set 90-70-90 targets to be achieved by 2030 [5].

India contributed to 7% of the global cancer incidence and 24% of global HPV related cancer incidence in 2020 [6]. Four out of five cervical cancers reported in India were caused by infections from HPV types 16 and 18 [7, 8]. Further, opportunistic screening of common cancers including cervical and oral cancers is being implemented in health facilities in India as a cancer control strategy [9]. Monitoring the impact of these primary and secondary prevention interventions can be done using the population-based and hospital-based cancer registries in India. Comprehensive understanding of the epidemiology of HPV related cancers would aid in defining the strategy for preventive interventions in the country. Hence, the current study describes the epidemiology of HPV related cancers, utilising the data from the Population Based Cancer Registries (PBCRs) and Hospital Based Cancer Registries (HBCRs) under the network of the National Cancer Registry Programme (NCRP) in India [10, 11].

## Methods

Based on the monograph findings from the International Agency for Research on Cancer (IARC) and the International Classification of Diseases, Tenth Revision, HPV related cancers are defined as the cancers of cervix uteri (C53), vulva (C51), vagina (C52), penis (C60), anus (C21) and oropharynx (C09-C10), oral cavity (C01-C06) and larynx (C32) [3, 12]. The current study presents the descriptive epidemiology of these HPV related cancers excluding oral cavity and larynx as the Population Attributable Fraction (PAF) of HPV infection to these sites is minimum (PAF: oral cavity 1.4% and larynx 1.1%, respectively) [4].

### Data sources

The NCRP comprises a network of several HBCRs and 38 PBCRs functioning under the Indian Council of Medical Research (ICMR) - National Centre for Disease Informatics and Research (NCDIR) at Bengaluru, India. PBCRs actively collect cancer incidence and mortality data on cancer patients residing for minimum of 1 year prior to diagnosis in the defined geographical area, whereas HBCRs collate information on clinical diagnosis, treatment modalities and outcome from the cancer patients availing treatment and care from various departments in the hospital from any part of the country. The cancer data collected by the NCRP network undergoes rigorous quality checks as per IARC norms. The present analysis utilised the finalised data on HPV related cancer sites reported from 28 PBCRs between 2012 and 2016 and 96 HBCRs between 2012 and 2019 [10, 11].

### Statistical analysis

Five-year age specific population size by sex for the years 2012–2016 was estimated using the 2001 and 2011 India Census data [13, 14]. The following measures were presented using the PBCR data: (a) crude incidence rate (CR) per 100,000 population, (b) age adjusted incidence rate (AAR) per 100,000 population using the World Population Standard [15] and (c) cumulative risk, which indicates the likelihood of developing cancer in the age group of 0–74 years. Joinpoint regression analysis was performed and annual percentage change (APC) in AARs was estimated using Joinpoint trend analysis software by the National Cancer Institute [16]. The projected incidence of HPV related cancers in India for the year 2025 was estimated and presented as number of cases. The clinical extent of the disease collected from the HBCRs was classified as localised (cancer restricted to the primary site), locoregional (locally advanced/lymph nodes) and distant metastasis and presented according to type of treatment modality.

## Results

There were 415,194 (males: 50.2%, females: 49.8%) incident cancer cases for all sites reported from 28 PBCRs between 2012 and 2016 in India and, 7.5% (males: 3.3%, females: 11.8%) were from all the HPV related sites. HBCR data of 2012–2019 reported 64,043 HPV related cancer cases (Supplementary Table 1).

### Incidence of HPV related cancers

For all the HPV related cancers, East Khasi Hills district, Meghalaya reported the highest age adjusted incidence rate (AAR) among males (AAR: 12.4 per 100,000) with highest cumulative risk of 1 in 69 men developing HPV related cancers between 0 and 74 years, followed by Kamrup urban PBCR, Assam (AAR: 10.7) and Meghalaya state (AAR: 9.1). Papumpare district in Arunachal Pradesh showed highest incidence (AAR: 34.5 per 100,000) among females, with lowest being Dibrugarh district, Assam (AAR: 6.6 per 100,000). The risk of developing HPV related cancer was lowest in Sikkim state among males (cumulative risk: 1 in 749) and Dibrugarh district among females (cumulative risk: 1 in 133) (Table 1).

### Cervical cancer

**Figure 1.**
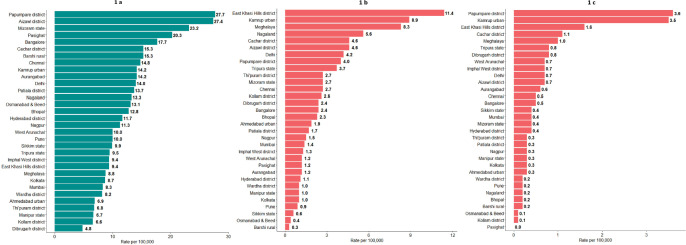
Age adjusted incidence rates in 28 PBCRs under NCRP. (a): Cervical cancer (C53), (b): Oropharyngeal cancer (C09-10) - males and (c): Oropharyngeal cancer (C09-10) – females.

**Figure 2.**
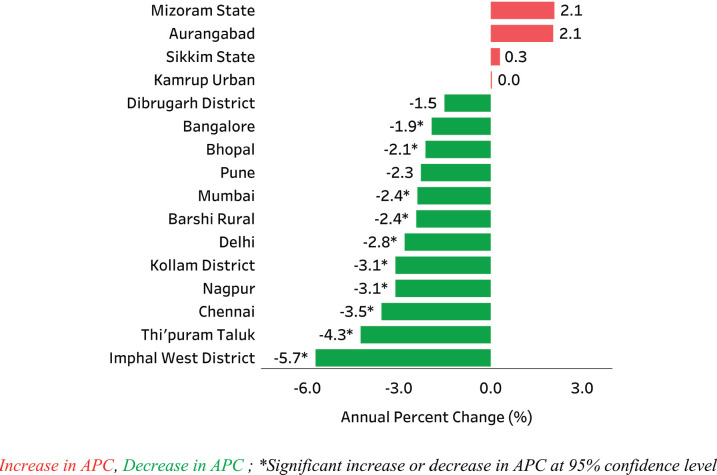
Annual Percent Change (APC) in age adjusted incidence rates (AARs) over the time period for cervical cancer (C53).

**Figure 3.**
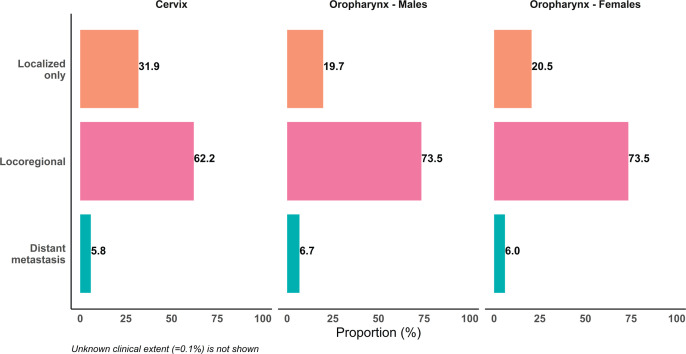
Proportion of patients according to the clinical extent of cancer for cervical (C53) and oropharyngeal cancer (C09-10) by gender reported in 96 HBCRs.

**Figure 4.**
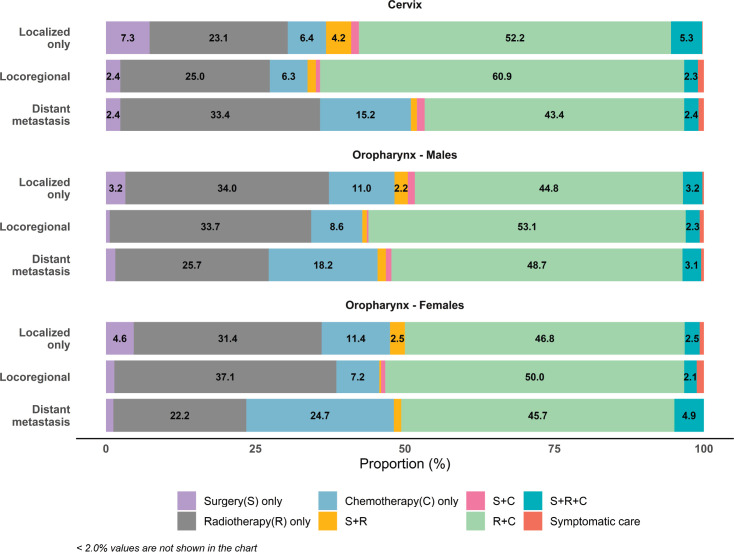
Proportion (%) of cancer patients according to type of treatment and clinical extent of the cancer for cervical (C53) and oropharyngeal cancer (C09-10) by gender reported in 96 HBCRs.

Nine out of ten HPV related cancers were cervical cancers in India (Supplementary Table 1). Papumpare district and Aizawl district in north eastern India reported the highest AAR of 27.7 and 27.4 per 100,000 women and lowest AAR (4.8 per 100,000) in Dibrugarh district, Assam (Figure 1a). Significant decrease in incidence rate was observed in 10 out of 16 PBCRs, with highest decrease (APC: −5.7) in Imphal West District, Manipur and no change in Kamrup urban, Assam (APC: 0.0) (Figure 2). Majority (62.2%) of the cervical cancers were presented with locoregional extent of cancer at the time of diagnosis (Figure 3). The most common mode of treatment was combination of radiotherapy and chemotherapy for all stages of cancer (Figure 4). Majority of the cervical cancers were squamous cell carcinomas (89.5%), followed by adenocarcinoma (6.6%) (Supplementary Table 2a).

### Oropharyngeal cancer

Oropharyngeal cancer was the most common among HPV related cancers in males (63.2%). Among females, they constituted 3.6% to HPV related cancers in India (Supplementary Table 1). East Khasi Hills district PBCR in Meghalaya reported the highest AAR (11.4 per 100,000), followed by Kamrup Urban PBCR (8.9 per 100,000) among males, whereas Papumpare district in Arunachal Pradesh (AAR: 3.6 per 100,000) and Kamrup Urban, Assam (AAR: 3.5 per 100,000) PBCRs had the highest incidence among females. The lowest incidence was observed in Barshi rural PBCR in Maharashtra among males (AAR: 0.3 per 100,000) and in Pasighat among females (AAR: <0.1 per 100,000) (Figure 1b and c). More than two-thirds of the patients were presented with locoregional spread of cancer at time of diagnosis (Figure 3). Two out of ten oropharyngeal cancer patients were presented at localised stage and majority of them were treated either with radiotherapy only or with combination of radiotherapy and chemotherapy (Figure 4). Most (86.3%) of the oropharyngeal cancers were squamous cell carcinomas (Supplementary Table 2b).

### Other HPV related cancers

One out of four HPV related cancers among males in India was penile cancers. Among females, the sites oropharynx, anus & anal canal, vulva and vagina together constituted 12.4% of HPV related cancers (Supplementary Table 1).

### Projection of HPV related cancer incidence for 2025

The projected number of incident HPV related cancers is 121,302 in 2025, contributing 7.7% (males: 3.3% and females: 12.0%) to total cancer cases in India. The leading HPV related sites would be oropharynx (60.6%), followed by penis (26.3%) among males and cervix uteri (88.4%) among females. The remaining sites, namely, anus & anal canal, vulva and vagina together would constitute 11%–13% of total HPV related cancers (Table 2).

## Discussion

This comprehensive study provides the incidence rates, cumulative risk, clinical extent of disease, treatment modality, histological classification of HPV related cancers in India from a large network of cancer registries. The study showed that cervical and oropharyngeal cancers were the most common HPV related cancers among females and males, respectively, in India. These cancers together contributed around 85.0% of total HPV related cancers.

Cervical cancer incidence was higher among PBCRs in north eastern region of India. However, its incidence rate showed a decreasing trend among most of the PBCRs over time [17]. The study found that most of the cervical cancers were diagnosed at locally advanced stage. Late diagnosis of cervical cancers could be attributed to low level of knowledge and awareness on risk factors, screening, as well as poor screening and early detection coverage among Indian women [18–20].

A combination of radiotherapy and chemotherapy treatment was advised irrespective of the stage of disease with varied proportions of surgery. The treatment pattern observed in this study aligned with the existing evidence [21–23]. A multi-centric cervical cancer study from India showed that 5-year survival was significantly higher with chemoradiation compared to radiotherapy alone in locoregional stage [24]. HPV infection was the main cause for cervical cancers with PAF ranging between 93% and 100% in India [25]. Examination of cervical cancer cases from central India revealed that HPV types 16, 18 and 45 were predominant types [26]. The progression of cervical cancer from initial HPV infection is modified by other factors such as age at menarche, age at marriage, sexual habits, high parity, tobacco use, prolonged use of oral contraceptives and co-infections (particularly Human Immunodeficiency Virus) [27]. Hence, prevention of cervical cancer includes screening, vaccination and behaviour change interventions.

The study found that six out of ten HPV related cancers among males were oropharyngeal cancers in India. A global study in 2017 attributed 22.0% (95% (confidence interval) CI: 5.0–44.0) of oropharyngeal cancer incidence in India to HPV infection [4]. However, recent studies among Indian population reported wide variations of HPV prevalence among oropharyngeal cancer cases ranging between 0.0% in south India and 44.4% in north, followed by west (26.7%) with HPV 16 and 58 as the predominant genotypes [28–33]. Reported variations in HPV prevalence among cancer cases in India are due to different detection methods, population, geographic locations and sample size. However, major proportion could be attributed to tobacco use (59.0%) and consumption of alcohol (4.7%) [34, 35]. Existing policies and programmes to prevent and control these risk factors should be reinforced to bring down cancer burden [36]. Stage at diagnosis (locoregional stage) and treatment provided (chemoradiation) were found to be in accordance with the existing evidence [37]. Also, a multi-institution study on survival of head and neck cancers showed that 5–year survival was significantly higher with stage I and II than Stage III and IV for oropharyngeal cancer [38].

Vulval and vaginal cancers are rare cancers that showed minor decline in incidence for longer term in India [39]. HPV infection was more common among young women leading to basaloid/warty lesions in vulva. The PAF to HPV infection by anus and anal canal (100%), vaginal cancer (78.0%; 95% CI: 68.0–86.0) and vulval cancer (30.3%; 95% CI: 25.3–35.0) was found to be high compared to laryngeal cancer (1.1; 95% CI: 0.1–5.1) and oral cavity cancer (1.4% 95% CI: 0.4–3.2) [1, 4].

Penile and anal cancer together constitutes 1.2% of total cancer cases in India. Globally, more than half (51.0%) of the penile cancers were attributed to HPV infection with PAF ranging between 47.0% and 55.0% [25]. This indicates a projected 3,337 HPV attributable penile cancers for 2025 in India. HPV 16, 18 and 33 are the predominant genotypes in anal, vulvar, vaginal and penile cancers [40–42]. Although India contributes to around 10% to total anus and anal cancer cases globally, trend analysis indicates stability in the incidence of anus and anal cancer for India [43].

HPV vaccines were licensed in 2008 for adolescent girls in India to be used on prescription base. Further, efforts were taken through demonstration vaccination projects, opportunistic screening and population-based screening [44–47]. However, poor screening coverage, low level of knowledge and awareness on risk factors, screening, HPV infection and vaccination among Indian women are hindering the cervical cancer control [8, 19, 48]. Growing population, ageing and changing sexual behaviour might avert the declining incidence trend in the absence of interventions as experienced in other settings [49]. It is estimated that in a medium Human Development Index country like India, efforts for scaling up HPV vaccination covering 80%–100% of target population together with two cervical cancer screenings per lifetime would help in achieving cervical cancer elimination by 2065-70 [50].

### Strengths and limitations

The main strength of the study is the use of high-quality data from HBCRs and PBCRs under the NCRP that are spread across the country. The data were abstracted from multiple sources such as cancer care hospitals, laboratories, and vital statistics departments using well trained registry staff, and cleaned using a robust quality check mechanism. Although the NCRP provides reliable information on incidence and treatment of malignant cancers related to HPV, they do not collect data pertaining to HPV genotype status and other risk factors.

## Conclusion

One out of 13 cancers in India is from HPV related sites. Implementation of effective prevention and control strategies like HPV vaccination and scaling up of screening, risk factor reduction could reduce the burden of HPV related cancers. Evidence from the NCRP serves as the baseline to monitor the impact of HPV policies and programmes in improving the outcome and prognosis.

## List of abbreviations

AAR, Age adjusted incidence rate; APC, Annual percentage change; CR, Crude incidence rate; HBCR, Hospital Based Cancer Registry; HPV, Human papillomavirus; IARC, International Agency for Research on Cancer; ICMR, Indian Council of Medical Research; NCDIR, National Centre for Disease Informatics and Research; NCRP, National Cancer Registry Programme; PBCR, Population Based Cancer Registry

## Conflicts of interest

The authors declare no conflicts of interest.

## Funding statement

The authors received no financial support for authorship of this article.

## Authorship contribution

Thilagavathi Ramamoorthy: Conceptualisation, methodology, formal statistical analysis, writing – original draft, review and editing. Sathish Kumar Krishnan: Methodology, formal statistical analysis, data curation, writing – review and editing. Priyanka Das: Data curation, interpretation, writing – review and editing. Kondalli Lakshminarayana Sudarshan: Data curation, interpretation, writing – review and editing. Prashant Mathur: Conceptualisation, methodology, investigation, writing – review and editing, visualization, supervision, project administration. All authors have read and provided approval to the final version of the manuscript. The work reported in the paper has been performed by the authors, unless clearly specified in the text.

## Ethical approval

This study was approved by the Institutional Ethics Committee of ICMR-NCDIR. Approval no: NCDIR/IEC/ 3052/2022.

## Figures and Tables

**Supplementary Table 1.** Number and proportion of HPV related cancers in India.

Sites of cancer	PBCR 2012–2016				HBCR 2012–2019			
	Males		Females		Males		Females	
	Number	%	Number	%	Number	%	Number	%
Oropharynx (C09-C10)	4,378	2.1	876	0.4	9,565	3.0	1,367	0.5
Anus & anal canal (C21)	867	0.4	567	0.3	1,893	0.6	1,193	0.4
Vulva (C51)	-	-	696	0.3	-	-	1,112	0.4
Vagina (C52)	-	-	878	0.4	-	-	1,749	0.6
Cervix uteri (C53)	-	-	21,354	10.3	-	-	44,300	15.2
Penis (C60)	1,683	0.8	-	-	2,864	0.9	-	-
Total HPV related cancers	6,928	3.3	24,371	11.8	14,322	4.5	49,721	17.1
Others	201477	96.7	182418	88.2	304776	95.5	241265	82.9
All sites (C00-97)	208,405	100.0	206,789	100.0	319,098	100.0	290,986	100.0

**Supplementary Table 2a.** Proportion according to the broad histological classification - Cervical cancer (C53).

Broad histological classification	*n*	%
**Squamous cell carcinoma**		
**Squamous cell carcinoma, NOS**	26,524	60.1
**Squamous cell carcinoma, non-keratinising**	8,314	18.9
**Squamous cell carcinoma, keratinising**	4,184	9.5
**All other squamous cell carcinomas**	430	1.0
**Adenocarcinoma**		
**Adenocarcinoma, NOS**	2,215	5.0
**All other adenocarcinomas**	687	1.6
**Carcinoma, NOS**	1,150	2.6
**Adenosquamous carcinoma**	426	1.0
**Mesenchymal tumours**		
**Sarcoma**	52	0.1
**Others**	120	0.3
**Total**	44,102	100.0

NOS, Not Otherwise Specified.

**Supplementary Table 2b.** Proportion according to the broad histological classification - Oropharyngeal cancer (C09-10).

Broad histological classification	Males		Females		Both genders	
	*n*	%	*n*	%	*n*	%
Epithelial cell tumours						
Squamous cell carcinoma, NOS	8,272	86.8	1,122	82.4	9,394	86.3
Squamous cell carcinoma, keratinising	261	2.7	48	3.5	309	2.8
Squamous cell carcinoma, non-keratinising	154	1.6	50	3.7	204	1.9
Verrucous carcinoma	6	0.1	2	0.1	8	0.1
All other squamous cell carcinomas	40	0.4	5	0.4	45	0.4
Adenocarcinoma	56	0.6	16	1.2	72	0.7
Mesenchymal tumours						
Sarcoma	7	0.1	4	0.3	11	0.1
Carcinoma, NOS	361	3.8	64	4.7	425	3.9
Others	370	3.9	51	3.7	421	3.8
Total	9,527	100.0	1,362	100.0	10,889	100.0

NOS, Not Otherwise Specified.

**Table 1.** Incidence of HPV related cancers registered in PBCRs under NCRP in 2012–2016.

Name of the PBCR	Males					Females				
	Number of HPV cancers	CR	AAR	Cumulative risk	Proportion to all sites of cancer (%)	Number of HPV cancers	CR	AAR	Cumulative risk	Proportion to all sites of cancer (%)
Delhi	1,218	4.4	5.9	1 in 135	3.9	3,329	13.7	16.1	1 in 54	11.5
Patiala district	205	3.9	4.1	1 in 211	3.8	732	15.4	15.0	1 in 60	12.0
Hyderabad district	144	2.3	2.8	1 in 300	2.8	672	11.4	14.2	1 in 58	10.4
Kollam district	270	4.3	3.4	1 in 231	2.7	673	9.6	7.1	1 in 119	6.9
Thi'puram district	347	4.4	3.5	1 in 235	2.6	914	10.5	7.8	1 in 108	6.4
Bangalore	374	2.7	3.5	1 in 228	2.8	2,122	16.8	19.8	1 in 44	13.4
Chennai	537	4.5	4.4	1 in 199	3.7	2,141	17.9	17.0	1 in 49	12.7
Kolkata	231	2.4	2.0	1 in 415	2.3	1,065	12.3	10.3	1 in 84	11.6
Ahmedabad urban	456	2.8	3.1	1 in 267	3.1	1,249	8.5	8.6	1 in 102	11.3
Aurangabad	52	1.6	2.0	1 in 422	2.7	413	13	15.7	1 in 54	20.6
Osmanabad & Beed	162	1.7	1.8	1 in 482	4.5	1,264	15	14.1	1 in 62	28.3
Barshi rural	23	1.7	1.6	1 in 594	3.2	227	18.7	16.9	1 in 51	27.9
Mumbai	627	2.2	2.6	1 in 321	2.4	2,350	10.1	9.9	1 in 87	8.6
Pune	235	1.7	2.1	1 in 400	2.4	1,273	9.8	11.1	1 in 78	11.8
Wardha district	99	3.0	2.6	1 in 307	4.1	325	10.3	8.9	1 in 102	12.8
Bhopal	124	2.9	3.6	1 in 226	3.5	471	11.9	14.2	1 in 58	13.1
Nagpur	217	3.4	3.4	1 in 249	3.6	839	12.9	12.5	1 in 71	13.9
Manipur state	112	1.5	2.0	1 in 399	3.0	494	6.3	7.8	1 in 109	11.0
Imphal West district	37	2.7	3.1	1 in 251	3.3	150	10.7	11.1	1 in 77	10.0
Mizoram state	91	3.1	4.1	1 in 206	2.1	603	20.6	24.3	1 in 41	16.1
Aizawl district	52	4.9	6.1	1 in 138	2.4	294	27.1	28.8	1 in 36	15.5
Sikkim state	17	1.0	1.2	1 in 749	1.5	134	8.9	11.4	1 in 76	11.8
Tripura state	383	3.9	4.7	1 in 182	5.8	921	9.7	10.9	1 in 82	18.7
West Arunachal	30	1.4	2.6	1 in 377	2.5	153	7.2	11.7	1 in 76	13.1
Papumpare district	14	2.8	5.3	1 in 166	3.0	81	16.1	34.5	1 in 23	15.3
Meghalaya	250	5.0	9.1	1 in 92	5.3	334	6.6	10.6	1 in 86	11.8
East Khasi Hills district	166	7.5	12.4	1 in 69	5.8	180	8	11.6	1 in 79	10.4
Nagaland	76	4.0	7.1	1 in 110	5.4	175	10	14.7	1 in 64	17.6
Pasighat	7	1.9	2.6	1 in 320	2.2	60	17.5	22.1	1 in 45	19.8
Cachar district	208	4.4	5.9	1 in 142	4.5	696	15.3	17.9	1 in 53	17.7
Dibrugarh district	109	3.1	4.0	1 in 220	4.3	187	5.5	6.6	1 in 133	8.4
Kamrup urban	324	9.9	10.7	1 in 80	5.2	555	17.4	19.4	1 in 45	11.6

HPV, Human papillomavirus; CR, Crude incidence rate per 100,000; AAR, Age adjusted incidence rate per 100,000; NCRP, National Cancer Registry Programme; Thi’puram, Thiruvananthapuram; PBCR, Population Based Cancer Registry

**Table 2.** Projected number of HPV related cancer incidence for India in 2025.

Site of cancer	Males		Females	
	Number	%	Number	%
Oropharynx	15,051	2.0	2,866	0.4
Anus & anal canal	3,260	0.4	2,301	0.3
Vulva	-		2,433	0.3
Vagina	-		3,606	0.4
Cervix uteri	-		85,241	10.6
Penis	6,544	0.9	-	-
Total HPV related cancers	24,855	3.3	96,447	12.0
Other sites	738,720	96.7	709,771	88.0
All sites (C00-C97)	763,575	100.0	806,218	100.0
